# Crystal structure and Hirshfeld surface analysis of 5-acetyl-2-amino-4-(4-bromo­phen­yl)-6-oxo-1-phenyl-1,4,5,6-tetra­hydro­pyridine-3-carbo­nitrile

**DOI:** 10.1107/S2056989022001232

**Published:** 2022-02-03

**Authors:** Ibrahim G. Mamedov, Victor N. Khrustalev, Mehmet Akkurt, Anton P. Novikov, Ayten R. Asgarova, Khatira N. Aliyeva, Anzurat A. Akobirshoeva

**Affiliations:** a Department of Chemistry, Baku State University, 23 Z. Khalilov str., Az, 1148 Baku, Azerbaijan; b Peoples’ Friendship University of Russia (RUDN University), 6 Miklukho-Maklay str., Moscow, 117198, Russian Federation; c N. D. Zelinsky Institute of Organic Chemistry RAS, Leninsky Prosp. 47, 119991 Moscow, Russian Federation; d Department of Physics, Faculty of Sciences, Erciyes University, 38039 Kayseri, Turkey; e Acad. Sci. Republ. Tajikistan, Kh. Yu. Yusufbekov Pamir Biol. Inst., 1 Kholdorova str., 736002, Khorog, Gbao, Tajikistan

**Keywords:** crystal structure, tetra­hydro­pyridine, hydrogen bonds, dimers, C—Br⋯π contacts, Hirshfeld surface analysis

## Abstract

The crystal structure of the title compound, C_20_H_16_BrN_3_O_2_, was determined using an inversion twin. Its asymmetric unit comprises two crystallographically independent mol­ecules (*A* and *B*) being the stereoisomers. Both mol­ecules are linked by pairs of N—H⋯O hydrogen bonds, forming a dimer with an *R* ^2^_2_(16) ring motif. The dimers are connected by further N—H⋯O and N—H⋯N hydrogen bonds, forming chains along the *c-*axis direction·C—Br⋯π inter­actions between these chains contribute to the stabilization of the mol­ecular packing. Hirshfeld surface analysis showed that the most important contributions to the crystal packing are from H⋯H, C⋯H/H⋯C, O⋯H/H⋯O, Br⋯H/H⋯Br and N⋯H/H⋯N inter­actions.

## Abstract 2

In the crystal, N—H⋯O hydrogen bonds link the mol­ecules into dimers with an 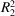 (16) ring motif. Further N—H⋯O and N—H⋯N hydrogen bonds connect the dimers into chains along the *c-*axis direction. C—Br⋯π and C=O⋯π inter­actions stabilize the mol­ecular packing, resulting in a three-dimensional network.

## Chemical context

Nitro­gen-based heterocycles are an important class of organic mol­ecules that are used extensively in different branches of chemistry (Yadigarov *et al.*, 2009 ▸; Abdelhamid *et al.*, 2011 ▸; Magerramov *et al.*, 2018 ▸; Yin *et al.*, 2020 ▸; Khalilov *et al.*, 2021 ▸). In particular, the synthesis of heterocyclic systems comprising a bioactive pyridine core with a broad spectrum of biological activities is noteworthy (Mamedov *et al.*, 2020 ▸; Wojcicka & Redzicka, 2021 ▸). On the other hand, the pyridine ring is an essential part of diverse natural products, such as nicotinic acid, nicotinamide, vitamin B_3_ and diverse alkaloids (Aida *et al.*, 2009 ▸). In the framework of our ongoing structural studies (Safarova *et al.*, 2019 ▸; Naghiyev *et al.*, 2020 ▸, 2021 **a* ▸,*b* ▸;* Maharramov *et al.*, 2021 ▸), we report here the crystal structure and Hirshfeld surface analysis of the title compound, 5-acetyl-2-amino-4-(4-bromo­phen­yl)-6-oxo-1-phenyl-1,4,5,6-tetra­hydro­pyridine-3-carbo­nitrile. 
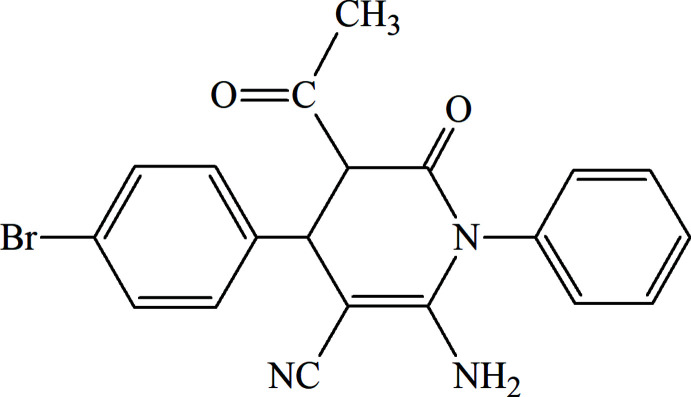


## Structural commentary

**Figure 1:**
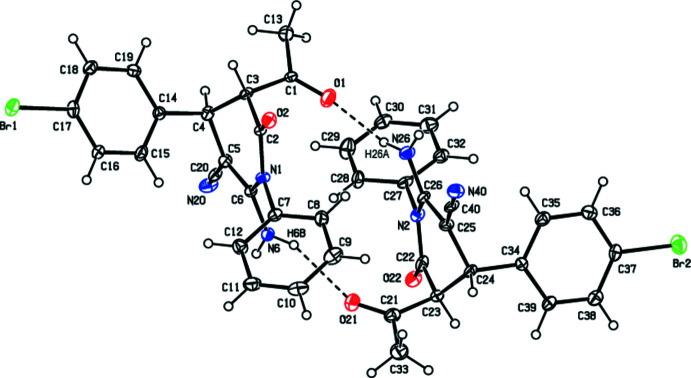
Asymmetric unit of the title compounds showing two crystallographically independent mol­ecules, *A* and *B*. Displacement ellipsoids are drawn at the 30% probability level. The inter­molecular N—H⋯O hydrogen bonds are drawn with dashed lines.

The title compound crystallizes in the monoclinic space group *Pc* with *Z* = 4, and with two mol­ecules, *A* and *B*, in the asymmetric unit (Fig. 1 ▸). These mol­ecules are stereoisimers with an *R*,*R* absolute configurations at C3 and C4 in mol­ecule *A*, whereas the corresponding atoms in *B*, C23 and C24, have an *S* configuration. In both mol­ecules, the conformation of the central di­hydro­pyridine ring is close to screw-boat [the puckering parameters (Cremer & Pople, 1975 ▸) are θ = 63.9 (11)°, φ = 148.9 (12)° in *A* and θ = 115.1 (11)°, φ = 339.4 (12)° in *B*]. In mol­ecule *A*, the phenyl (C7–C12) and bromo­phenyl (C14–C19) rings form dihedral angles of 64.0 (4) and 86.3 (4)°, respectively, with the mean plane of the central di­hydro­pyridine ring. In mol­ecule *B*, the corresponding dihedral angles are 77.2 (4) and 83.9 (4)°. The acetyl groups in both mol­ecules are almost planar [largest deviations of 0.005 (8) and 0.035 (8) Å for atoms C1 (*A*) and C23 (*B*), respectively] and they make the dihedral angles of 89.5 (5) and 87.7 (5)° with the mean planes of the di­hydro­pyridine rings in these mol­ecules.

## Supra­molecular features

**Figure 2:**
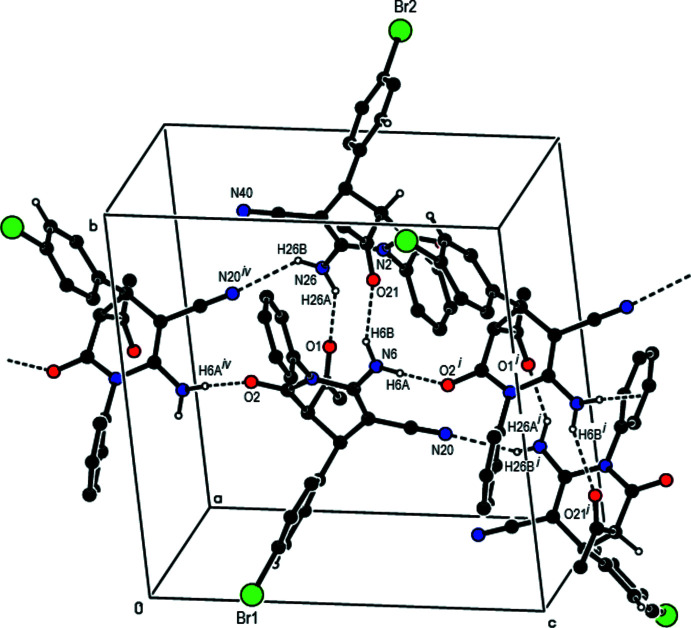
A general view of the N—H⋯O and N—H⋯N hydrogen bonds in the structure of the title compound.

**Figure 6:**
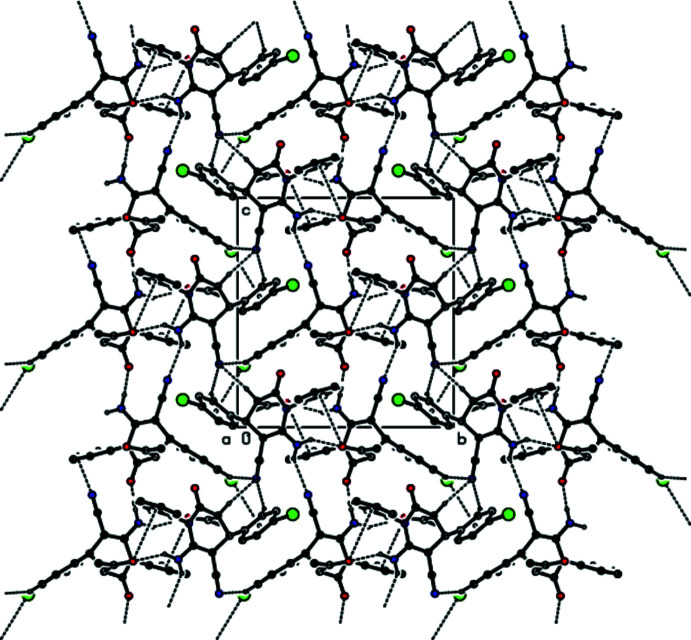
The C—Br⋯π and C=O⋯π inter­actions in the structure of the title compound viewed down the *a* axis.

Strong N6—H6*B*⋯O21 and N26—H26*A*⋯O1 hydrogen bonds (Fig. 1 ▸, Table 1 ▸) link mol­ecules *A* and *B* into dimers with an 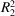 (16) ring motif (Bernstein *et al.*, 1995 ▸). These dimers are additionally stabilized by C=O⋯π inter­actions [O21⋯*Cg*2 = 3.620 (8) Å, C21=O21⋯*Cg*2 = 110.8 (6)°, O1⋯*Cg*5 = 3.748 (8) Å, C1=O1⋯*Cg*5 = 125.1 (6)°, where *Cg*2 and *Cg*5 are the centroids of the C7–C12 phenyl ring in mol­ecule *A* and the C27–C32 phenyl ring in mol­ecule *B*, respectively]. The dimers are connected by N—H⋯O and N—H⋯N hydrogen bonds with an 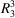 (14) ring motif into chains along the *c-*axis direction (Table 1 ▸; Figs. 2 ▸, 3 ▸, 4 ▸ and 5 ▸). C—Br⋯π inter­actions are also observed [Br1⋯*Cg*6*^v^ =* 3.407 (4) Å, C17—Br1⋯*Cg*6*^v^ =* 145.2 (3)°; symmetry code (v) −1 + *x*, 1 − *y*, − 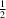 + *z*; *Cg*6 is the centroid of the C34–C39 ring]. Together with the other inter­molecular contacts given in Table 2 ▸, these inter­actions contribute to the stabilization of the mol­ecular packing, forming a three-dimensional network (Figs. 6 ▸ and 7 ▸).

## Hirshfeld surface analysis

**Figure 8:**
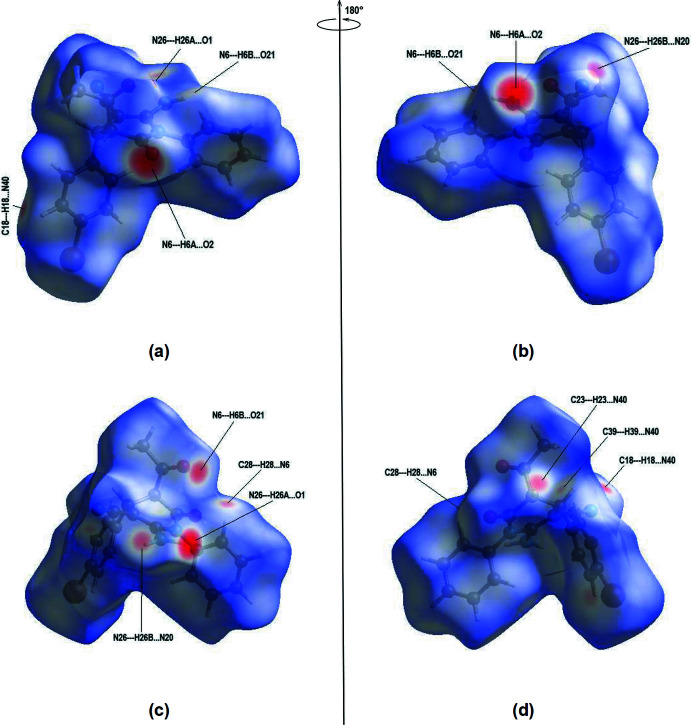
Front and back views of the Hirshfeld surfaces mapped over *d*_norm_ for mol­ecule *A* (*a*, *b*) and mol­ecule *B* (*c*, *d*).

To visualize the inter­molecular inter­actions for both independent mol­ecules *A* and *B*, *CrystalExplorer17* (Turner *et al.*, 2017 ▸) was used to generate Hirshfeld surfaces and corresponding two-dimensional fingerprint plots. The *d*_norm_ mappings were performed in the range of −0.6596 to 1.4042 arbitrary units for mol­ecule *A* and −0.5436 to 1.4926 arbitrary units for mol­ecule *B*. Bright red circles on the *d*_norm_ surfaces (Fig. 8 ▸ *a*,*b*,*c*,*d*) indicate regions of N—H⋯O inter­actions. The N—H⋯N and C—H⋯N inter­actions (Tables 1 ▸ and 2 ▸) also cause red spots on the Hirshfeld surfaces.

**Figure 9:**
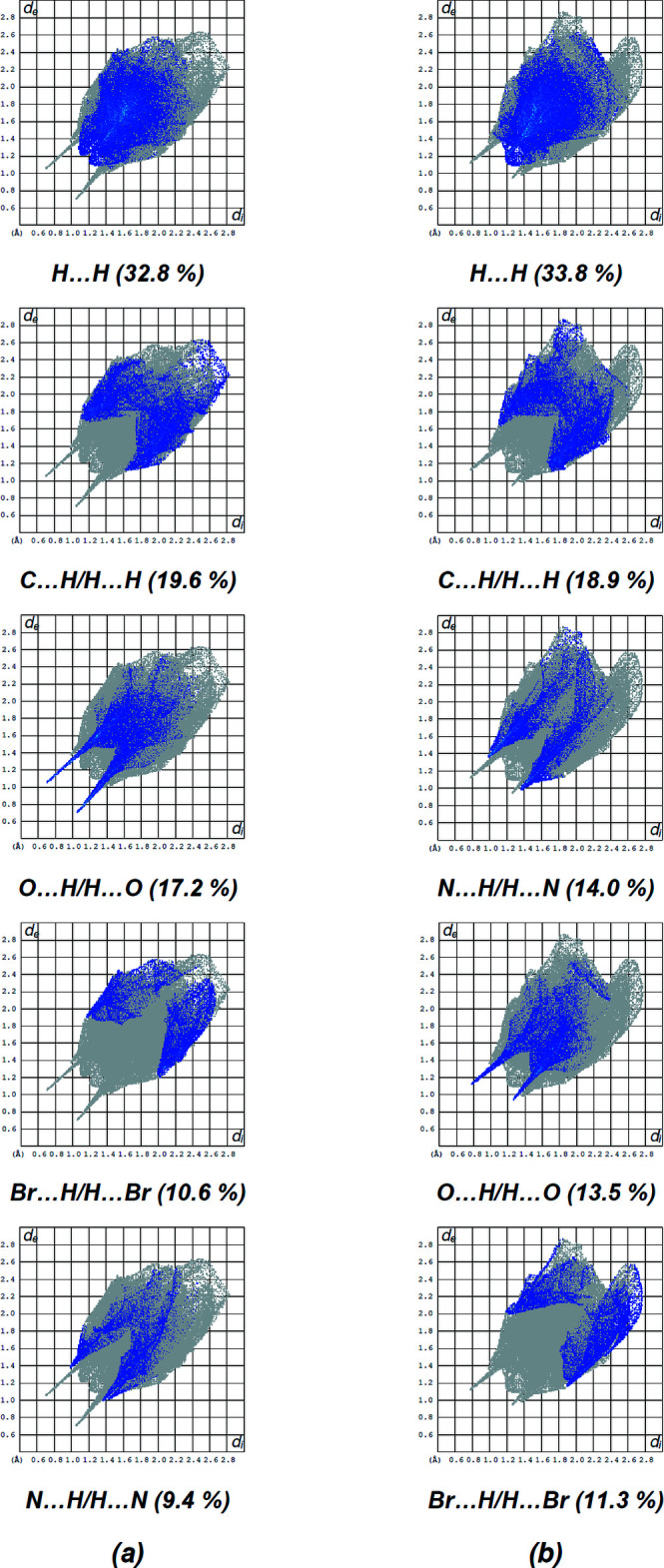
The two-dimensional fingerprint plots [(*a*) for mol­ecule *A* and (*b*) for mol­ecule *B*], showing all inter­actions and those delineated into H⋯H, C⋯H/H⋯C, O⋯H/H⋯O, Br⋯H/H⋯Br, N⋯H/H⋯N inter­actions. The *d*_i_ and *d*_e_ values are the closest inter­nal and external distances (in Å) from given points on the Hirshfeld surfaces.

The fingerprint plots (Fig. 9 ▸) reveal that while the H⋯H inter­actions make the greatest contributions (Table 3 ▸), as would be expected for a mol­ecule with such a predominance of H atoms, C⋯H/H⋯C, O⋯H/H⋯O, Br⋯H/H⋯Br and N⋯H/H⋯N contacts are also substantial. Table 3 ▸ gives the contributions of the other, less significant contacts. The fact that the same type of inter­actions provide different contributions to the Hirshfeld surface for mol­ecules *A* and *B* can be attributed to the different environments of these mol­ecules in the crystalline state.

## Database survey

A search of the Cambridge Structural Database (CSD, Version 5.42, update of September 2021; Groom *et al.*, 2016 ▸) for the tetra­hydro­pyridine unit gave 1340 hits, and some of which, namely OZAKOS (Naghiyev *et al.*, 2021*c* ▸), JEBREQ (Mohana *et al.*, 2017 ▸), JEBRAM (Mohana *et al.*, 2017 ▸), SETWUK (Suresh *et al.*, 2007 ▸) and SETWOE (Suresh *et al.*, 2007 ▸) closely resemble the title compound.

In OZAKOS (space group: *Pc*), the mol­ecular conformation of the title compound is stabilized by an intra­molecular O—H⋯O hydrogen bond, forming an *S*(6) ring motif. In the crystal, mol­ecules are linked by inter­molecular N—H⋯N and C—H⋯N hydrogen bonds, and N—H⋯π and C—H⋯π inter­actions, forming a three-dimensional network.

In both the related salts, JEBREQ (space group: *P* 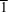 ) and JEBRAM (space group: *P* 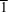 ), the N atom in the 1-position of the pyrimidine ring is protonated. In the hydrated salt JEBREQ, the presence of the water mol­ecule prevents the formation of the familiar 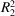 (8) ring motif. Instead, an expanded ring [*i.e. R* ^3^_2_(8)] is formed involving the sulfonate group, the pyrimidinium cation and the water mol­ecule. Both salts form a supra­molecular homosynthon [ 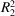 (8) ring motif] through N—H⋯N hydrogen bonds. The mol­ecular structures are further stabilized by π–π stacking, and C=O⋯π, C—H⋯O and C—H⋯Cl inter­actions. It appears that the protonation state of the pyrimidine ring influences the inter­molecular inter­actions within the crystal lattice to a substantial extent. In JEBRAM, the protonated N atom and the amino group of the pyrimidinium cation inter­act with the carboxyl­ate group of the anion through N—H⋯O hydrogen bonds, forming a heterosynthon with an 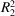 (8) ring motif.

The polysubstituted pyridines, SETWUK (space group: *P*2_1_/*n*) and SETWOE (space group: *P*2_1_/*c*), adopt nearly planar structures. The crystal structure of SETWUK is stabilized by inter­molecular C—H⋯F and C—H⋯π inter­actions. The C—H⋯F bond generates a linear chain with a *C*(14) motif. The crystal structure of SETWOE is stabilized by inter­molecular C—H⋯O and C—H⋯π inter­actions. The C—H⋯O hydrogen bonds generate rings with *R^2^_2_* (14) and *R^2^_2_* (20) motifs. In addition, in SETWOE and SETWUK, intra­molecular O—H⋯O inter­actions are found, which generate an *S*(6) graph-set motif. No significant ar­yl–aryl or π–π inter­actions exist in these structures. All this bears some resemblance to the title compound.

## Synthesis and crystallization

To a solution of 2-(4-bromo­benzyl­idene)malono­nitrile (1.19 g; 5.1 mmol) and acetoacetanilide (0.92 g; 5.2 mmol) in methanol (25 mL), piperidine (2–3 drops) was added and the mixture was stirred at room temperature for 48 h. Then 15 mL of methanol were removed by rotary evaporation from the reaction mixture, which was left overnight. The precipitated crystals were separated by filtration and recrystallized from ethanol/water (1:1) solution (yield 66%; m.p. 536–537 K).

^1^H NMR (300 MHz, DMSO-*d*_6_, m.h.): 2.29 (*s*, 3H, CH_3_—C=O); 4.15 (*d*, 1H, CH-Ar); 4.34 (*d*, 1H, CH—C=O); 5.98 (*s*, 2H, NH_2_); 7.12–7.35 (*m*, 5H, 5CH_ar_); 7.40 (*d*, 2H, 2CH_ar_); 7.61 (*d*, 2H, 2CH_ar_).

^13^C NMR (75 MHz, DMSO-*d*_6_, m.h.): 27.86 (CH_3_—C=O), 37.94 (CH—Ar), 57.24 (=C_quat_), 62.41 (CH—C=O), 117.21 (CN), 121.25 (Br-Car), 127.67 (CH_ar_), 128.19 (2CH_ar_), 129.58 (2CH_ar_), 130.15 (2CH_ar_), 130.74 (2CH_ar_), 136.98 (C_ar_), 140.37 (C_ar_), 154.14 (=C_quat_), 166.20 (N—C=O), 202.55 (C=O).

## Refinement details

Crystal data, data collection and structure refinement details are summarized in Table 4 ▸. All H atoms were positioned geometrically (N—H = 0.90 Å, C—H = 0.95–1.00 Å) and refined as riding with *U*_iso_(H) = 1.2*U*_eq_(C, N) or 1.5*U*_eq_(C-meth­yl).

## Supplementary Material

Crystal structure: contains datablock(s) I. DOI: 10.1107/S2056989022001232/yk2165sup1.cif

Structure factors: contains datablock(s) I. DOI: 10.1107/S2056989022001232/yk2165Isup2.hkl

Supporting information file. DOI: 10.1107/S2056989022001232/yk2165Isup3.cml

CCDC reference: 2149629

Additional supporting information:  crystallographic information; 3D view; checkCIF report

## Figures and Tables

**Figure 3:**
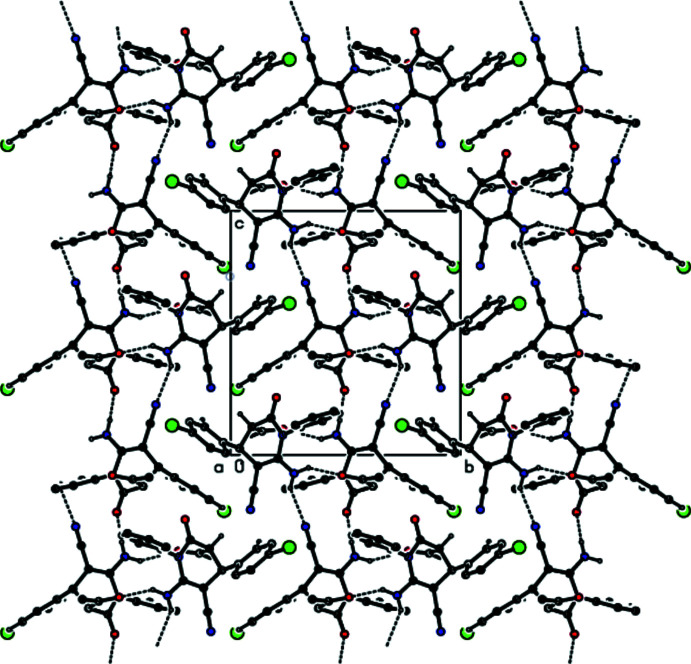
The crystal packing of the title compound viewed down the *a* axis, showing chains running along the *c-*axis direction formed through N—H⋯O and N—H⋯N hydrogen bonds.

**Figure 4:**
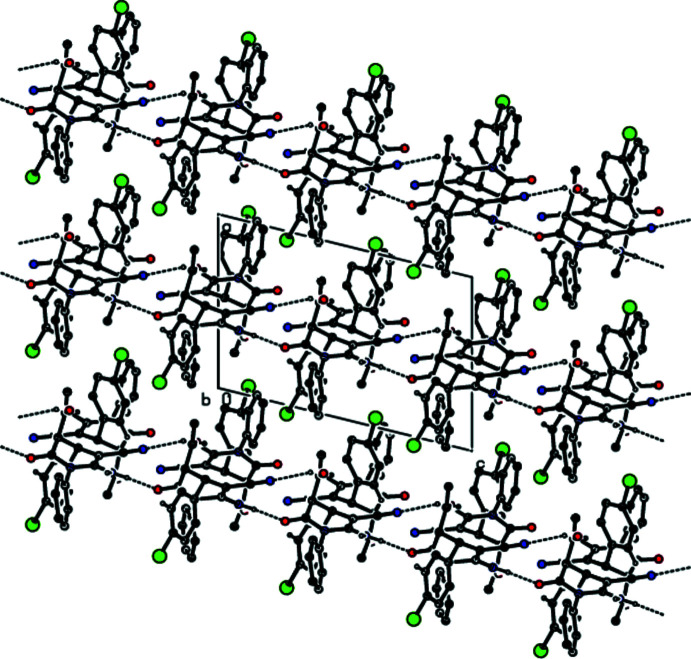
The crystal packing of the title compound viewed down the *b* axis, showing chains running along the *c* axis formed through N—H⋯O and N—H⋯N hydrogen bonds.

**Figure 5:**
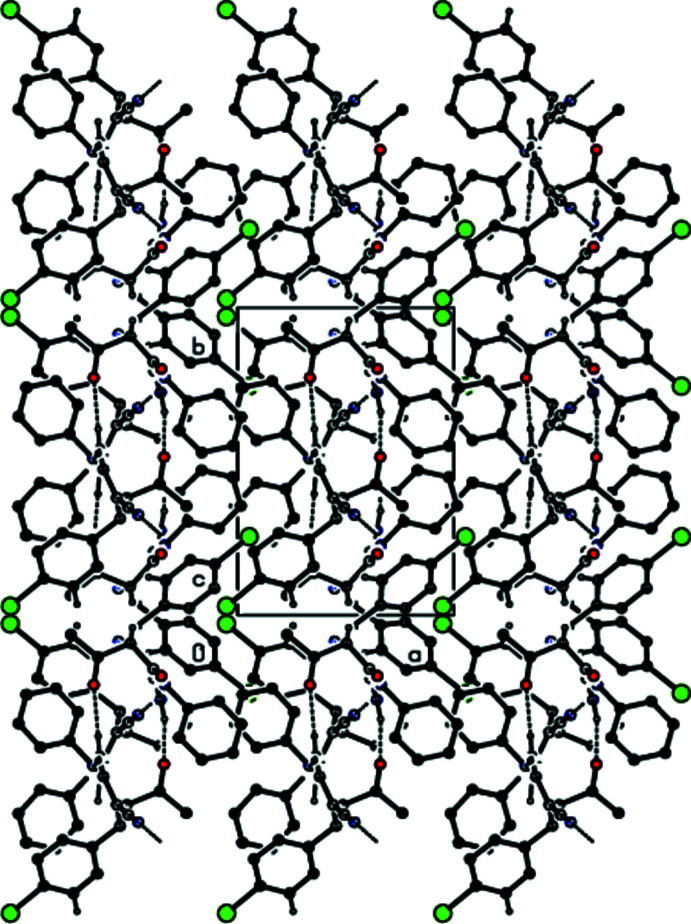
The crystal packing of the title compound viewed down the *c* axis, with inter­molecular N—H⋯O, C—H⋯N and N—H⋯N hydrogen bonds.

**Figure 7:**
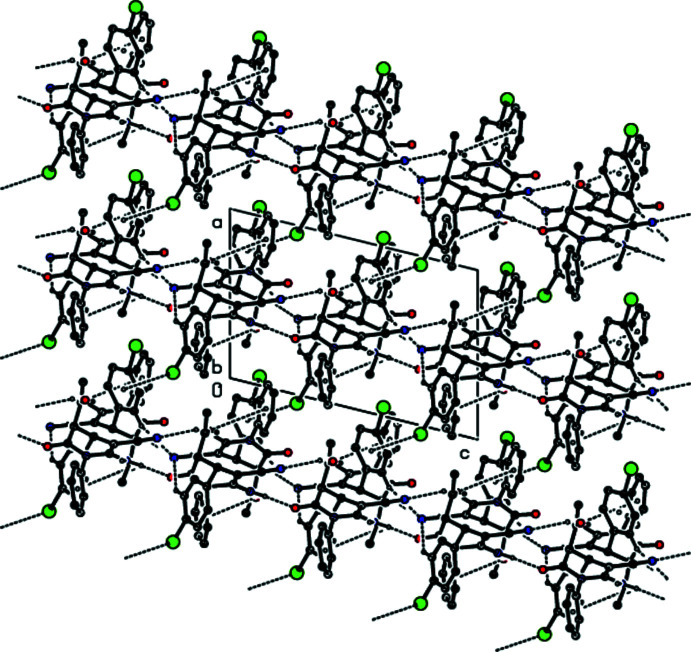
A view of the C—Br⋯π and C=O⋯π inter­actions in the structure of the title compound viewed down the *b* axis.

**Table 1:** Hydrogen-bond geometry (Å, °)

*D*—H⋯*A*	*D*—H	H⋯*A*	*D*⋯*A*	*D*—H⋯*A*
N6—H6*A*⋯O2^i^	0.90	1.87	2.766 (9)	175
N6—H6*B*⋯O21	0.90	2.31	3.115 (9)	149
C18—H18⋯N40^ii^	0.95	2.46	3.256 (12)	141
C23—H23⋯N40^iii^	1.00	2.47	3.426 (11)	161
N26—H26*A*⋯O1	0.90	1.99	2.784 (9)	146
N26—H26*B*⋯N20^iv^	0.90	2.43	3.139 (10)	136

Symmetry codes: (i) 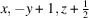 ; (ii) 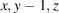 ; (iii) 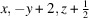 ; (iv) 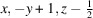 .

**Table 2:** Summary of short inter­atomic contacts (Å) in the title compound

Contact	Distance	Symmetry operation
O2⋯H30	2.63	*x* − 1, −*y* + 1, *z* − 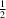
O1⋯H26*A*	1.99	*x*, *y*, *z*
H13*C*⋯H16	2.46	*x* + 1, *y*, *z*
O2⋯H6*A*	1.87	*x*, −*y* + 1, *z* − 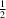
H18⋯N40	2.46	*x*, *y* − 1, *z*
N20⋯H26*B*	2.43	*x*, −*y* + 1, *z* + 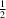
C9⋯Br2	3.377 (10)	*x* − 1, −*y* + 2, *z* − 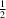
H13*C*⋯O22	2.79	*x*, −*y* + 1, *z* − 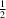
C16⋯H36	2.86	*x* − 1, *y* − 1, *z*
H11⋯H26*A*	2.47	*x* − 1, *y*, *z*
O21⋯H31	2.84	*x* − 1, *y*, *z*
H23⋯N40	2.47	*x*, −*y* + 2, *z* + 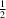
H31⋯O21	2.84	*x* + 1, *y*, *z*

**Table 3:** Percentage contributions of inter­atomic contacts to the Hirshfeld surfaces of mol­ecules *A* and *B* of the title compound

Contact	Contribution for *A*	Contribution for *B*
H⋯H	32.8	33.8
C⋯H/H⋯C	19.6	18.9
O⋯H/H⋯O	17.2	13.5
Br⋯H/H⋯Br	10.6	11.3
N⋯H/H⋯N	9.4	14.0
Br⋯C/C⋯Br	4.8	4.6
N⋯O/O⋯N	2.1	–
C⋯O/O⋯C	1.4	1.3
Br⋯O/O⋯Br	0.8	0.9
C⋯C	0.7	0.7
N⋯N	0.5	0.4
Br⋯N/N⋯Br	0.1	0.6

**Table 4:** Experimental details

Crystal data	
Chemical formula	C_20_H_16_BrN_3_O_2_
*M*_r_	410.26
Crystal system, space group	Monoclinic, *P* *c*
Temperature (K)	100
*a*, *b*, *c* (Å)	9.5889 (7), 13.2144 (10), 14.4529 (10)
β (°)	103.9395 (18)
*V* (Å^3^)	1777.4 (2)
*Z*	4
Radiation type	Mo *K*α
μ (mm^−1^)	2.33
Crystal size (mm)	0.05 × 0.04 × 0.03

Data collection	
Diffractometer	Bruker D8 QUEST PHOTON-III CCD
Absorption correction	Multi-scan (*SADABS*; Krause *et al.*, 2015 ▸)
*T*_min_, *T*_max_	0.818, 0.926
No. of measured, independent and observed [*I* > 2σ(*I*)] reflections	34410, 10756, 5403
*R*_int_	0.099
(sin θ/λ)_max_ (Å^−1^)	0.714

Refinement	
*R*[*F* ^2^ > 2σ(*F* ^2^)], *wR*(*F* ^2^), *S*	0.065, 0.132, 0.98
No. of reflections	10756
No. of parameters	471
No. of restraints	2
H-atom treatment	H-atom parameters constrained
Δρ_max_, Δρ_min_ (e Å^−3^)	0.50, −0.66
Absolute structure	Refined as an inversion twin
Absolute structure parameter	0.473 (14)

Computer programs: *CrysAlis PRO* (Rigaku OD, 2021 ▸), *SHELXT* (Sheldrick, 2015*a* ▸), *SHELXL* (Sheldrick, 2015*b* ▸), *ORTEP-3 for Windows* (Farrugia, 2012 ▸) and *PLATON* (Spek, 2020 ▸).

## supplementary crystallographic information

### Crystal data

**Table:**

C_20_H_16_BrN_3_O_2_	*F*(000) = 832
*M**_r_* = 410.26	*D*_x_ = 1.533 Mg m^−^^3^
Monoclinic, *P**c*	Mo *K*α radiation, λ = 0.71073 Å
*a* = 9.5889 (7) Å	Cell parameters from 3126 reflections
*b* = 13.2144 (10) Å	θ = 2.7–24.0°
*c* = 14.4529 (10) Å	µ = 2.33 mm^−^^1^
β = 103.9395 (18)°	*T* = 100 K
*V* = 1777.4 (2) Å^3^	Prism, colourless
*Z* = 4	0.05 × 0.04 × 0.03 mm

### Data collection

**Table:**

Bruker D8 QUEST PHOTON-III CCD diffractometer	5403 reflections with *I* > 2σ(*I*)
φ and ω scans	*R*_int_ = 0.099
Absorption correction: multi-scan (SADABS; Krause *et al.*, 2015)	θ_max_ = 30.5°, θ_min_ = 2.1°
*T*_min_ = 0.818, *T*_max_ = 0.926	*h* = −13→13
34410 measured reflections	*k* = −18→18
10756 independent reflections	*l* = −20→20

### Refinement

**Table:**

Refinement on *F*^2^	Hydrogen site location: mixed
Least-squares matrix: full	H-atom parameters constrained
*R*[*F*^2^ > 2σ(*F*^2^)] = 0.065	*w* = 1/[σ^2^(*F*_o_^2^) + (0.0401*P*)^2^] where *P* = (*F*_o_^2^ + 2*F*_c_^2^)/3
*wR*(*F*^2^) = 0.132	(Δ/σ)_max_ < 0.001
*S* = 0.98	Δρ_max_ = 0.50 e Å^−^^3^
10756 reflections	Δρ_min_ = −0.66 e Å^−^^3^
471 parameters	Extinction correction: SHELXL, Fc^*^=kFc[1+0.001xFc^2^λ^3^/sin(2θ)]^-1/4^
2 restraints	Extinction coefficient: 0.0039 (3)
Primary atom site location: difference Fourier map	Absolute structure: Refined as an inversion twin
Secondary atom site location: difference Fourier map	Absolute structure parameter: 0.473 (14)

### Special details

**Table:**

Geometry. All esds (except the esd in the dihedral angle between two l.s. planes) are estimated using the full covariance matrix. The cell esds are taken into account individually in the estimation of esds in distances, angles and torsion angles; correlations between esds in cell parameters are only used when they are defined by crystal symmetry. An approximate (isotropic) treatment of cell esds is used for estimating esds involving l.s. planes.
Refinement. Refined as a two-component inversion twin.

### Fractional atomic coordinates and isotropic or equivalent isotropic displacement parameters (Å^2^)

**Table:**

	*x*	*y*	*z*	*U*_iso_*/*U*_eq_	
Br1	−0.05184 (9)	0.02892 (6)	0.27044 (7)	0.0328 (2)	
N1	0.3304 (7)	0.5094 (5)	0.4197 (5)	0.0194 (15)	
C1	0.6385 (9)	0.4260 (6)	0.3992 (6)	0.0216 (19)	
O1	0.6581 (7)	0.5145 (5)	0.4155 (5)	0.0456 (19)	
C2	0.3768 (9)	0.4655 (6)	0.3455 (6)	0.0219 (18)	
O2	0.3284 (7)	0.4954 (4)	0.2642 (4)	0.0273 (14)	
C3	0.4864 (9)	0.3816 (6)	0.3708 (6)	0.0211 (18)	
H3	0.4792	0.3389	0.3127	0.025*	
C4	0.4547 (9)	0.3140 (6)	0.4498 (6)	0.0221 (19)	
H4	0.5405	0.2699	0.4736	0.026*	
C5	0.4402 (9)	0.3820 (6)	0.5310 (6)	0.0229 (19)	
C6	0.3781 (9)	0.4741 (6)	0.5141 (6)	0.0213 (18)	
N6	0.3596 (7)	0.5398 (5)	0.5816 (5)	0.0219 (15)	
H6A	0.3529	0.5257	0.6413	0.026*	
H6B	0.3534	0.6055	0.5646	0.026*	
C7	0.2213 (9)	0.5898 (6)	0.3983 (6)	0.0212 (18)	
C8	0.2595 (10)	0.6844 (7)	0.3737 (7)	0.031 (2)	
H8	0.3538	0.6970	0.3663	0.037*	
C9	0.1581 (10)	0.7610 (7)	0.3600 (7)	0.036 (2)	
H9	0.1814	0.8267	0.3416	0.044*	
C10	0.0223 (11)	0.7409 (8)	0.3732 (7)	0.036 (2)	
H10	−0.0463	0.7940	0.3661	0.044*	
C11	−0.0144 (11)	0.6464 (7)	0.3963 (6)	0.035 (2)	
H11	−0.1089	0.6334	0.4031	0.042*	
C12	0.0861 (10)	0.5691 (7)	0.4099 (6)	0.030 (2)	
H12	0.0620	0.5030	0.4269	0.036*	
C13	0.7611 (10)	0.3551 (6)	0.4072 (7)	0.034 (2)	
H13A	0.7314	0.2868	0.4207	0.050*	
H13B	0.8413	0.3770	0.4591	0.050*	
H13C	0.7915	0.3546	0.3472	0.050*	
C14	0.3267 (9)	0.2446 (6)	0.4069 (6)	0.0213 (17)	
C15	0.1891 (10)	0.2670 (6)	0.4115 (7)	0.029 (2)	
H15	0.1720	0.3262	0.4445	0.034*	
C16	0.0740 (10)	0.2057 (6)	0.3695 (6)	0.029 (2)	
H16	−0.0213	0.2229	0.3717	0.034*	
C17	0.1025 (10)	0.1185 (6)	0.3240 (6)	0.026 (2)	
C18	0.2390 (10)	0.0940 (6)	0.3159 (7)	0.029 (2)	
H18	0.2561	0.0350	0.2828	0.035*	
C19	0.3494 (10)	0.1581 (6)	0.3576 (6)	0.026 (2)	
H19	0.4441	0.1428	0.3525	0.031*	
C20	0.4925 (10)	0.3499 (6)	0.6274 (7)	0.024 (2)	
N20	0.5390 (9)	0.3255 (6)	0.7052 (5)	0.0335 (19)	
Br2	1.05193 (11)	1.25495 (8)	0.62011 (8)	0.0409 (3)	
N2	0.6616 (7)	0.7734 (5)	0.5811 (5)	0.0220 (16)	
C21	0.3521 (10)	0.8636 (7)	0.5959 (6)	0.028 (2)	
O21	0.3329 (8)	0.7728 (5)	0.6051 (5)	0.0403 (18)	
C22	0.6085 (10)	0.8245 (6)	0.6505 (6)	0.027 (2)	
O22	0.6450 (7)	0.8015 (4)	0.7343 (4)	0.0305 (15)	
C23	0.5025 (9)	0.9077 (6)	0.6148 (6)	0.0238 (19)	
H23	0.5119	0.9588	0.6670	0.029*	
C24	0.5288 (9)	0.9623 (6)	0.5266 (6)	0.0226 (19)	
H24	0.4424	1.0048	0.4993	0.027*	
C25	0.5389 (9)	0.8820 (6)	0.4542 (6)	0.0230 (19)	
C26	0.6117 (9)	0.7941 (6)	0.4838 (6)	0.0210 (18)	
N26	0.6426 (8)	0.7245 (5)	0.4234 (5)	0.0279 (17)	
H26A	0.6602	0.6614	0.4466	0.034*	
H26B	0.6454	0.7409	0.3634	0.034*	
C27	0.7687 (10)	0.6958 (6)	0.6136 (6)	0.0234 (19)	
C28	0.7276 (10)	0.6009 (6)	0.6383 (7)	0.030 (2)	
H28	0.6293	0.5858	0.6336	0.036*	
C29	0.8324 (11)	0.5287 (7)	0.6698 (7)	0.040 (3)	
H29	0.8056	0.4632	0.6865	0.048*	
C30	0.9761 (11)	0.5507 (6)	0.6776 (7)	0.033 (2)	
H30	1.0476	0.5007	0.6998	0.040*	
C31	1.0139 (11)	0.6444 (7)	0.6530 (7)	0.035 (2)	
H31	1.1123	0.6590	0.6575	0.042*	
C32	0.9120 (10)	0.7187 (6)	0.6216 (6)	0.028 (2)	
H32	0.9398	0.7842	0.6059	0.034*	
C33	0.2270 (11)	0.9361 (7)	0.5651 (8)	0.041 (3)	
H33A	0.2541	1.0027	0.5936	0.061*	
H33B	0.1440	0.9103	0.5862	0.061*	
H33C	0.2021	0.9422	0.4955	0.061*	
C34	0.6606 (8)	1.0327 (6)	0.5511 (6)	0.0188 (17)	
C35	0.7743 (10)	1.0222 (7)	0.5076 (6)	0.028 (2)	
H35	0.7720	0.9690	0.4630	0.034*	
C36	0.8913 (10)	1.0875 (7)	0.5276 (7)	0.029 (2)	
H36	0.9687	1.0787	0.4980	0.035*	
C37	0.8931 (10)	1.1659 (6)	0.5917 (6)	0.026 (2)	
C38	0.7802 (10)	1.1788 (6)	0.6350 (6)	0.027 (2)	
H38	0.7820	1.2325	0.6789	0.033*	
C39	0.6654 (10)	1.1133 (6)	0.6139 (6)	0.025 (2)	
H39	0.5874	1.1232	0.6429	0.030*	
C40	0.4888 (10)	0.8997 (6)	0.3553 (7)	0.025 (2)	
N40	0.4482 (10)	0.9142 (5)	0.2749 (6)	0.0307 (16)	

### Atomic displacement parameters (Å^2^)

**Table:**

	*U*^11^	*U*^22^	*U*^33^	*U*^12^	*U*^13^	*U*^23^
Br1	0.0341 (5)	0.0228 (4)	0.0388 (5)	−0.0033 (5)	0.0036 (4)	−0.0037 (5)
N1	0.027 (4)	0.020 (3)	0.012 (3)	0.005 (3)	0.007 (3)	0.002 (3)
C1	0.027 (5)	0.016 (4)	0.025 (5)	0.002 (4)	0.013 (4)	−0.003 (3)
O1	0.030 (4)	0.026 (4)	0.081 (5)	−0.002 (3)	0.013 (4)	−0.012 (4)
C2	0.025 (5)	0.017 (4)	0.026 (5)	−0.001 (4)	0.013 (4)	−0.005 (4)
O2	0.035 (4)	0.025 (3)	0.021 (3)	−0.001 (3)	0.006 (3)	0.004 (3)
C3	0.028 (5)	0.018 (4)	0.018 (4)	0.001 (4)	0.006 (4)	−0.005 (3)
C4	0.031 (5)	0.019 (4)	0.015 (4)	−0.002 (4)	0.004 (4)	0.001 (3)
C5	0.031 (5)	0.021 (4)	0.018 (4)	−0.001 (4)	0.008 (4)	0.001 (3)
C6	0.026 (5)	0.020 (4)	0.020 (4)	−0.004 (4)	0.010 (4)	−0.006 (4)
N6	0.031 (4)	0.021 (4)	0.015 (4)	−0.003 (3)	0.008 (3)	−0.001 (3)
C7	0.024 (5)	0.020 (4)	0.019 (4)	0.004 (3)	0.003 (4)	0.000 (3)
C8	0.027 (5)	0.023 (4)	0.043 (6)	−0.001 (4)	0.010 (5)	0.002 (4)
C9	0.039 (6)	0.031 (5)	0.035 (6)	0.012 (5)	0.000 (5)	0.003 (4)
C10	0.036 (6)	0.042 (6)	0.030 (6)	0.014 (5)	0.006 (5)	−0.005 (5)
C11	0.039 (6)	0.033 (5)	0.037 (6)	0.010 (5)	0.017 (5)	0.000 (4)
C12	0.026 (5)	0.030 (5)	0.033 (5)	0.002 (4)	0.008 (4)	0.005 (4)
C13	0.029 (5)	0.028 (5)	0.044 (6)	−0.001 (4)	0.010 (5)	−0.002 (4)
C14	0.024 (5)	0.019 (4)	0.021 (4)	0.000 (4)	0.006 (4)	0.002 (4)
C15	0.041 (6)	0.015 (4)	0.032 (5)	−0.002 (4)	0.012 (5)	0.000 (4)
C16	0.031 (5)	0.018 (4)	0.040 (6)	−0.003 (4)	0.014 (5)	−0.006 (4)
C17	0.027 (5)	0.025 (5)	0.023 (5)	−0.008 (4)	0.001 (4)	0.002 (4)
C18	0.031 (5)	0.020 (4)	0.037 (6)	0.002 (4)	0.008 (5)	−0.003 (4)
C19	0.022 (5)	0.030 (5)	0.029 (5)	−0.001 (4)	0.010 (4)	−0.007 (4)
C20	0.032 (5)	0.015 (4)	0.024 (5)	0.002 (4)	0.006 (4)	−0.001 (4)
N20	0.045 (5)	0.031 (4)	0.025 (5)	0.011 (4)	0.009 (4)	0.001 (3)
Br2	0.0417 (6)	0.0460 (6)	0.0350 (5)	−0.0143 (5)	0.0092 (4)	−0.0033 (5)
N2	0.020 (4)	0.017 (4)	0.026 (4)	0.006 (3)	0.000 (3)	0.002 (3)
C21	0.035 (6)	0.027 (5)	0.023 (5)	0.004 (4)	0.008 (4)	0.000 (4)
O21	0.051 (5)	0.026 (4)	0.048 (5)	−0.004 (3)	0.021 (4)	0.000 (3)
C22	0.038 (6)	0.016 (4)	0.025 (5)	−0.003 (4)	0.005 (4)	0.001 (4)
O22	0.044 (4)	0.025 (3)	0.023 (3)	0.003 (3)	0.008 (3)	0.003 (3)
C23	0.027 (5)	0.018 (4)	0.029 (5)	0.002 (4)	0.012 (4)	−0.003 (4)
C24	0.029 (5)	0.013 (4)	0.025 (5)	0.003 (4)	0.007 (4)	−0.001 (3)
C25	0.031 (5)	0.021 (4)	0.019 (4)	0.004 (4)	0.010 (4)	0.001 (4)
C26	0.028 (5)	0.013 (4)	0.023 (5)	0.001 (3)	0.007 (4)	−0.004 (3)
N26	0.034 (4)	0.022 (4)	0.030 (4)	0.006 (3)	0.011 (4)	0.000 (3)
C27	0.031 (5)	0.018 (4)	0.022 (5)	0.008 (4)	0.007 (4)	−0.001 (3)
C28	0.026 (5)	0.019 (4)	0.040 (6)	−0.002 (4)	0.003 (4)	0.006 (4)
C29	0.041 (6)	0.023 (5)	0.050 (6)	0.004 (5)	−0.001 (5)	0.012 (5)
C30	0.039 (6)	0.023 (5)	0.038 (6)	0.011 (4)	0.009 (5)	−0.001 (4)
C31	0.033 (6)	0.038 (6)	0.034 (6)	0.005 (4)	0.005 (5)	−0.003 (4)
C32	0.035 (5)	0.022 (4)	0.028 (5)	0.006 (4)	0.009 (4)	0.002 (4)
C33	0.039 (6)	0.035 (5)	0.050 (7)	0.006 (5)	0.015 (5)	0.004 (5)
C34	0.017 (4)	0.018 (4)	0.020 (4)	0.003 (3)	0.003 (3)	0.005 (3)
C35	0.037 (6)	0.022 (5)	0.025 (5)	0.003 (4)	0.009 (4)	0.001 (4)
C36	0.028 (5)	0.031 (5)	0.030 (5)	0.003 (4)	0.010 (4)	0.001 (4)
C37	0.031 (5)	0.020 (4)	0.026 (5)	−0.004 (4)	0.003 (4)	0.003 (4)
C38	0.039 (6)	0.024 (4)	0.018 (5)	0.000 (4)	0.005 (4)	0.000 (4)
C39	0.031 (5)	0.017 (4)	0.025 (5)	0.004 (4)	0.004 (4)	0.002 (4)
C40	0.036 (5)	0.013 (4)	0.028 (5)	0.008 (4)	0.012 (4)	0.000 (4)
N40	0.039 (4)	0.027 (4)	0.030 (4)	0.010 (4)	0.014 (3)	0.008 (4)

### Geometric parameters (Å, º)

**Table:**

Br1—C17	1.908 (9)	Br2—C37	1.890 (9)
N1—C2	1.383 (10)	N2—C26	1.400 (10)
N1—C6	1.410 (10)	N2—C22	1.403 (11)
N1—C7	1.470 (10)	N2—C27	1.447 (10)
C1—O1	1.199 (9)	C21—O21	1.226 (10)
C1—C13	1.486 (12)	C21—C33	1.516 (12)
C1—C3	1.533 (12)	C21—C23	1.517 (12)
C2—O2	1.220 (10)	C22—O22	1.215 (10)
C2—C3	1.511 (11)	C22—C23	1.501 (11)
C3—C4	1.537 (11)	C23—C24	1.538 (11)
C3—H3	1.0000	C23—H23	1.0000
C4—C5	1.510 (11)	C24—C25	1.509 (11)
C4—C14	1.538 (11)	C24—C34	1.541 (11)
C4—H4	1.0000	C24—H24	1.0000
C5—C6	1.351 (11)	C25—C26	1.369 (11)
C5—C20	1.428 (12)	C25—C40	1.414 (12)
C6—N6	1.349 (10)	C26—N26	1.350 (10)
N6—H6A	0.8999	N26—H26A	0.8993
N6—H6B	0.9000	N26—H26B	0.9000
C7—C8	1.374 (11)	C27—C32	1.384 (12)
C7—C12	1.374 (12)	C27—C28	1.387 (11)
C8—C9	1.385 (12)	C28—C29	1.380 (12)
C8—H8	0.9500	C28—H28	0.9500
C9—C10	1.387 (13)	C29—C30	1.386 (14)
C9—H9	0.9500	C29—H29	0.9500
C10—C11	1.361 (13)	C30—C31	1.362 (12)
C10—H10	0.9500	C30—H30	0.9500
C11—C12	1.386 (12)	C31—C32	1.382 (12)
C11—H11	0.9500	C31—H31	0.9500
C12—H12	0.9500	C32—H32	0.9500
C13—H13A	0.9800	C33—H33A	0.9800
C13—H13B	0.9800	C33—H33B	0.9800
C13—H13C	0.9800	C33—H33C	0.9800
C14—C15	1.370 (12)	C34—C35	1.391 (11)
C14—C19	1.391 (11)	C34—C39	1.392 (11)
C15—C16	1.386 (12)	C35—C36	1.389 (13)
C15—H15	0.9500	C35—H35	0.9500
C16—C17	1.386 (12)	C36—C37	1.386 (12)
C16—H16	0.9500	C36—H36	0.9500
C17—C18	1.380 (12)	C37—C38	1.385 (12)
C18—C19	1.376 (12)	C38—C39	1.375 (12)
C18—H18	0.9500	C38—H38	0.9500
C19—H19	0.9500	C39—H39	0.9500
C20—N20	1.151 (11)	C40—N40	1.149 (11)
			
C2—N1—C6	121.4 (7)	C26—N2—C22	121.9 (7)
C2—N1—C7	119.0 (7)	C26—N2—C27	120.6 (7)
C6—N1—C7	119.4 (7)	C22—N2—C27	117.5 (7)
O1—C1—C13	121.1 (8)	O21—C21—C33	121.4 (9)
O1—C1—C3	121.3 (8)	O21—C21—C23	121.1 (8)
C13—C1—C3	117.6 (7)	C33—C21—C23	117.4 (8)
O2—C2—N1	119.5 (7)	O22—C22—N2	121.6 (8)
O2—C2—C3	123.4 (7)	O22—C22—C23	122.3 (8)
N1—C2—C3	117.1 (7)	N2—C22—C23	116.1 (7)
C2—C3—C1	110.2 (6)	C22—C23—C21	108.4 (7)
C2—C3—C4	110.8 (7)	C22—C23—C24	113.3 (7)
C1—C3—C4	111.6 (7)	C21—C23—C24	111.6 (7)
C2—C3—H3	108.0	C22—C23—H23	107.8
C1—C3—H3	108.0	C21—C23—H23	107.8
C4—C3—H3	108.0	C24—C23—H23	107.8
C5—C4—C3	107.7 (6)	C25—C24—C23	107.2 (6)
C5—C4—C14	117.0 (7)	C25—C24—C34	113.4 (7)
C3—C4—C14	109.2 (7)	C23—C24—C34	112.5 (7)
C5—C4—H4	107.5	C25—C24—H24	107.8
C3—C4—H4	107.5	C23—C24—H24	107.8
C14—C4—H4	107.5	C34—C24—H24	107.8
C6—C5—C20	118.7 (8)	C26—C25—C40	118.6 (8)
C6—C5—C4	120.9 (8)	C26—C25—C24	119.5 (7)
C20—C5—C4	120.4 (7)	C40—C25—C24	121.5 (7)
N6—C6—C5	125.3 (8)	N26—C26—C25	123.4 (8)
N6—C6—N1	114.7 (7)	N26—C26—N2	116.2 (7)
C5—C6—N1	119.9 (7)	C25—C26—N2	120.3 (7)
C6—N6—H6A	127.7	C26—N26—H26A	116.2
C6—N6—H6B	115.8	C26—N26—H26B	121.5
H6A—N6—H6B	116.5	H26A—N26—H26B	122.3
C8—C7—C12	122.1 (8)	C32—C27—C28	120.9 (8)
C8—C7—N1	119.4 (8)	C32—C27—N2	119.0 (8)
C12—C7—N1	118.4 (7)	C28—C27—N2	120.0 (8)
C7—C8—C9	118.7 (9)	C29—C28—C27	118.7 (9)
C7—C8—H8	120.6	C29—C28—H28	120.6
C9—C8—H8	120.6	C27—C28—H28	120.6
C8—C9—C10	119.4 (9)	C28—C29—C30	120.8 (9)
C8—C9—H9	120.3	C28—C29—H29	119.6
C10—C9—H9	120.3	C30—C29—H29	119.6
C11—C10—C9	121.1 (9)	C31—C30—C29	119.4 (9)
C11—C10—H10	119.5	C31—C30—H30	120.3
C9—C10—H10	119.5	C29—C30—H30	120.3
C10—C11—C12	120.0 (10)	C30—C31—C32	121.3 (9)
C10—C11—H11	120.0	C30—C31—H31	119.3
C12—C11—H11	120.0	C32—C31—H31	119.3
C7—C12—C11	118.7 (9)	C31—C32—C27	118.8 (8)
C7—C12—H12	120.7	C31—C32—H32	120.6
C11—C12—H12	120.7	C27—C32—H32	120.6
C1—C13—H13A	109.5	C21—C33—H33A	109.5
C1—C13—H13B	109.5	C21—C33—H33B	109.5
H13A—C13—H13B	109.5	H33A—C33—H33B	109.5
C1—C13—H13C	109.5	C21—C33—H33C	109.5
H13A—C13—H13C	109.5	H33A—C33—H33C	109.5
H13B—C13—H13C	109.5	H33B—C33—H33C	109.5
C15—C14—C19	118.3 (8)	C35—C34—C39	117.7 (8)
C15—C14—C4	122.4 (7)	C35—C34—C24	121.7 (7)
C19—C14—C4	119.3 (7)	C39—C34—C24	120.5 (7)
C14—C15—C16	121.8 (8)	C36—C35—C34	121.7 (8)
C14—C15—H15	119.1	C36—C35—H35	119.2
C16—C15—H15	119.1	C34—C35—H35	119.2
C15—C16—C17	117.8 (9)	C37—C36—C35	118.8 (9)
C15—C16—H16	121.1	C37—C36—H36	120.6
C17—C16—H16	121.1	C35—C36—H36	120.6
C18—C17—C16	122.3 (8)	C38—C37—C36	120.6 (9)
C18—C17—Br1	118.6 (7)	C38—C37—Br2	120.1 (7)
C16—C17—Br1	119.1 (7)	C36—C37—Br2	119.3 (7)
C19—C18—C17	117.6 (8)	C39—C38—C37	119.5 (8)
C19—C18—H18	121.2	C39—C38—H38	120.2
C17—C18—H18	121.2	C37—C38—H38	120.2
C18—C19—C14	122.1 (8)	C38—C39—C34	121.7 (8)
C18—C19—H19	119.0	C38—C39—H39	119.2
C14—C19—H19	119.0	C34—C39—H39	119.2
N20—C20—C5	177.7 (10)	N40—C40—C25	180.0 (14)
			
C6—N1—C2—O2	176.9 (8)	C26—N2—C22—O22	175.0 (8)
C7—N1—C2—O2	1.3 (11)	C27—N2—C22—O22	−3.7 (12)
C6—N1—C2—C3	−3.0 (11)	C26—N2—C22—C23	−4.0 (11)
C7—N1—C2—C3	−178.6 (7)	C27—N2—C22—C23	177.3 (7)
O2—C2—C3—C1	94.4 (10)	O22—C22—C23—C21	−86.3 (10)
N1—C2—C3—C1	−85.7 (9)	N2—C22—C23—C21	92.7 (9)
O2—C2—C3—C4	−141.5 (8)	O22—C22—C23—C24	149.1 (8)
N1—C2—C3—C4	38.4 (10)	N2—C22—C23—C24	−31.9 (10)
O1—C1—C3—C2	13.1 (12)	O21—C21—C23—C22	−3.8 (12)
C13—C1—C3—C2	−167.8 (7)	C33—C21—C23—C22	176.2 (8)
O1—C1—C3—C4	−110.6 (10)	O21—C21—C23—C24	121.7 (9)
C13—C1—C3—C4	68.5 (10)	C33—C21—C23—C24	−58.3 (10)
C2—C3—C4—C5	−52.2 (9)	C22—C23—C24—C25	51.6 (9)
C1—C3—C4—C5	71.1 (9)	C21—C23—C24—C25	−71.1 (8)
C2—C3—C4—C14	75.8 (8)	C22—C23—C24—C34	−73.7 (9)
C1—C3—C4—C14	−160.9 (7)	C21—C23—C24—C34	163.5 (7)
C3—C4—C5—C6	36.6 (11)	C23—C24—C25—C26	−40.8 (10)
C14—C4—C5—C6	−86.8 (10)	C34—C24—C25—C26	83.9 (9)
C3—C4—C5—C20	−143.3 (8)	C23—C24—C25—C40	146.4 (8)
C14—C4—C5—C20	93.4 (10)	C34—C24—C25—C40	−88.9 (10)
C20—C5—C6—N6	−0.5 (14)	C40—C25—C26—N26	2.2 (13)
C4—C5—C6—N6	179.6 (8)	C24—C25—C26—N26	−170.8 (8)
C20—C5—C6—N1	177.6 (8)	C40—C25—C26—N2	−179.3 (8)
C4—C5—C6—N1	−2.3 (13)	C24—C25—C26—N2	7.7 (12)
C2—N1—C6—N6	161.5 (7)	C22—N2—C26—N26	−163.9 (7)
C7—N1—C6—N6	−22.9 (10)	C27—N2—C26—N26	14.7 (11)
C2—N1—C6—C5	−16.8 (12)	C22—N2—C26—C25	17.5 (12)
C7—N1—C6—C5	158.8 (8)	C27—N2—C26—C25	−163.9 (8)
C2—N1—C7—C8	−73.8 (10)	C26—N2—C27—C32	81.3 (10)
C6—N1—C7—C8	110.5 (9)	C22—N2—C27—C32	−100.0 (9)
C2—N1—C7—C12	110.5 (9)	C26—N2—C27—C28	−100.2 (10)
C6—N1—C7—C12	−65.2 (10)	C22—N2—C27—C28	78.4 (10)
C12—C7—C8—C9	−0.2 (14)	C32—C27—C28—C29	−1.0 (14)
N1—C7—C8—C9	−175.7 (8)	N2—C27—C28—C29	−179.4 (8)
C7—C8—C9—C10	1.3 (14)	C27—C28—C29—C30	0.6 (15)
C8—C9—C10—C11	−2.2 (15)	C28—C29—C30—C31	−0.5 (16)
C9—C10—C11—C12	2.0 (15)	C29—C30—C31—C32	0.8 (15)
C8—C7—C12—C11	0.0 (14)	C30—C31—C32—C27	−1.2 (14)
N1—C7—C12—C11	175.6 (8)	C28—C27—C32—C31	1.3 (13)
C10—C11—C12—C7	−0.9 (14)	N2—C27—C32—C31	179.7 (8)
C5—C4—C14—C15	25.4 (12)	C25—C24—C34—C35	1.3 (11)
C3—C4—C14—C15	−97.2 (9)	C23—C24—C34—C35	123.2 (8)
C5—C4—C14—C19	−157.7 (8)	C25—C24—C34—C39	177.4 (7)
C3—C4—C14—C19	79.7 (9)	C23—C24—C34—C39	−60.7 (10)
C19—C14—C15—C16	0.6 (13)	C39—C34—C35—C36	1.9 (13)
C4—C14—C15—C16	177.5 (8)	C24—C34—C35—C36	178.1 (8)
C14—C15—C16—C17	1.6 (13)	C34—C35—C36—C37	−0.9 (14)
C15—C16—C17—C18	−2.9 (14)	C35—C36—C37—C38	0.0 (13)
C15—C16—C17—Br1	177.1 (7)	C35—C36—C37—Br2	179.7 (7)
C16—C17—C18—C19	1.9 (14)	C36—C37—C38—C39	−0.1 (13)
Br1—C17—C18—C19	−178.0 (7)	Br2—C37—C38—C39	−179.8 (6)
C17—C18—C19—C14	0.4 (14)	C37—C38—C39—C34	1.2 (13)
C15—C14—C19—C18	−1.6 (13)	C35—C34—C39—C38	−2.0 (12)
C4—C14—C19—C18	−178.7 (8)	C24—C34—C39—C38	−178.3 (8)

### Hydrogen-bond geometry (Å, º)

**Table:**

*D*—H···*A*	*D*—H	H···*A*	*D*···*A*	*D*—H···*A*
N6—H6*A*···O2^i^	0.90	1.87	2.766 (9)	175
N6—H6*B*···O21	0.90	2.31	3.115 (9)	149
C18—H18···N40^ii^	0.95	2.46	3.256 (12)	141
C23—H23···N40^iii^	1.00	2.47	3.426 (11)	161
N26—H26*A*···O1	0.90	1.99	2.784 (9)	146
N26—H26*B*···N20^iv^	0.90	2.43	3.139 (10)	136

Symmetry codes: (i) *x*, −*y*+1, *z*+1/2; (ii) *x*, *y*−1, *z*; (iii) *x*, −*y*+2, *z*+1/2; (iv) *x*, −*y*+1, *z*−1/2.
